# Limonene Emissions: Do Different Types Have Different Biological Effects?

**DOI:** 10.3390/ijerph181910505

**Published:** 2021-10-07

**Authors:** Neda Nematollahi, Perran A. Ross, Ary A. Hoffmann, Spas D. Kolev, Anne Steinemann

**Affiliations:** 1 Department of Infrastructure Engineering, Melbourne School of Engineering, The University of Melbourne, Parkville, VIC 3010, Australia; anne.steinemann@unimelb.edu.au; 2 School of BioSciences and Bio21 Institute, Faculty of Science, The University of Melbourne, Parkville, VIC 3052, Australia; perran.ross@unimelb.edu.au (P.A.R.); ary@unimelb.edu.au (A.A.H.); 3 School of Chemistry, The University of Melbourne, Parkville, VIC 3010, Australia; s.kolev@unimelb.edu.au; 4 College of Science and Engineering, James Cook University, Townsville, QLD 4814, Australia

**Keywords:** limonene, orange oil, fragrance, fragranced consumer products, repellency, attraction, mosquitoes, air quality, health

## Abstract

Limonene is one of the most abundant pollutants indoors, and it contributes to the formation of additional pollutants, such as formaldehyde and photochemical smog. Limonene is commonly used in fragranced consumer products, such as cleaning supplies and air fresheners, which have also been associated with health problems. Limonene can exist in different enantiomeric forms (R-limonene and S-limonene) and be derived from different sources. However, little is known about whether different forms and sources of limonene may have different effects. This research explored whether different types of limonene, at the same concentrations, could elicit different biological effects. To investigate this question, the study employed *Aedes aegypti* mosquitoes, which have sophisticated olfactory abilities, in olfactometer tests of repellency/attraction. The results indicate that a synthetic source of R-limonene is more repellent than a natural source of R-limonene. In addition, synthetic sources of both R-limonene and S-limonene are not significantly different in repellency. These findings can contribute to our understanding and further exploration of the effects of a common fragrance compound on air quality and health.

## 1. Introduction

Chiral terpenes, such as limonene, are among the most abundant pollutants within indoor environments [1,2,3,4]. A primary contributor of limonene emissions indoors is fragranced consumer products, such as air fresheners, cleaning supplies, and personal care products [5,6,7,8]. Further, terpenes emitted from fragranced products can generate additional pollutants, such as formaldehyde and ultrafine particles indoors [9] and aerosols and photochemical smog outdoors [10]. 

Volatile emissions from fragranced consumer products have been associated with human health problems, such as migraine headaches and respiratory difficulties, in nearly one-third of the adult population across four countries [11]. According to chemical analyses of consumer products, limonene and other terpenes are present in fragranced products but absent in fragrance-free products [5,8]. Thus, limonene is of interest for its role as an air pollutant and its association with adverse health effects.

Limonene has a lemon or orange scent, and appears as two enantiomers: R-limonene and S-limonene. Limonene is commonly biosynthetized in nature, such as in oranges, and it can be chemically synthesized, such as for fragrance formulations. In biosynthesis, limonene appears predominantly as R-limonene, and in chemical synthesis, limonene can be produced as either R-limonene or S-limonene, or as a mixture of enantiomers.

The main component of orange oil is R-limonene, about 95%, and the other 5% consists of a different mixture of carbonyl components, waxes, and other terpenes, which vary for different kind of fruits [12,13]. The cold-press method is one preferred approach for the extraction of limonene from citrus. The solvent extraction method is another common approach. However, the use of solvents, such as hexane, methanol, ethanol, and acetone [14], can introduce petrochemical contaminants into the oils.

The olfactory system of insects can detect a wide range of volatile chemical stimuli to navigate their environment in search of food and oviposition sites [15]. Among different insect species, mosquitoes exhibit a particularly sophisticated olfactory ability [16,17]. Previous studies have used mosquitoes as bioindicators for toxicity [18,19,20] as well as indicators of environmental changes [21]. Insects have also been identified a promising model for pre-screening and toxicity testing [22]. 

The overall aim of this research is to explore whether different types (enantiomeric forms and sources) of limonene, at the same concentrations, may elicit different biological effects. The specific aims of this study are to investigate and compare the repellency effects of (1) R-limonene from a natural source and a synthetic source, and (2) R-limonene and S-limonene from synthetic sources. Using *Aedes aegypti* adult mosquitoes as a test species, the study employed controlled tests of repellency/attraction with different limonene samples emitted in an olfactometer. This study represents, to our knowledge, the first published inquiry into whether the same chiral molecule can elicit different biological effects depending on enantiomer or source (natural or synthetic).

## 2. Materials and Methods

### 2.1. R- and S-Limonene Reagents

The R- and S-limonene reagents, purchased from Sigma-Aldrich, Australia and containing 97% and 96% of the corresponding enantiomers, respectively, were used as received.

### 2.2. Natural Orange Oil Extraction

For this study, the cold-press method was used to extract orange oil from orange peel. Organic and unwaxed oranges were purchased from local stores in Melbourne, Australia. The orange peels were grated using a stainless steel grater to remove the coloured (flavedo) portion of orange peels. Then, grated orange peels were pureed along with a small amount of deionized water, using a kitchen blender allocated to solely this purpose. The mixture was filtered using a fabric netting to remove the oil-in-water emulsion from orange peel pulp. Finally, the orange peel oil was extracted by centrifugation of oil-in-water emulsion. The main volatile organic compounds (VOCs) in the orange oil and those of the R- and S-limonene reagents were identified by headspace GC/MS [7]. The concentration of R-limonene in the cold-press orange oil was determined by the same technique. 

### 2.3. Mosquito Strains and Colony Maintenance 

The study used a laboratory colony of *Aedes aegypti* mosquitoes for all experiments. Mosquitoes were collected as eggs from Cairns, Queensland in 2015 and reared in a temperature-controlled laboratory environment at 26 °C ± 1 °C with a 12 h photoperiod. Larvae were reared in trays filled with 4 L of reverse osmosis water at a controlled density of 450 larvae per tray. Larvae were fed tropical fish food tablets ad libitum until pupation. Female mosquitoes were blood fed on the forearms of human volunteers. Eggs were collected on sandpaper strips that were partially submerged in larval rearing water. Blood feeding of female mosquitoes on human volunteers for this research was approved by the University of Melbourne Human Ethics Committee (approval 0723847). All adult subjects provided informed written consent (no children were involved).

### 2.4. Repellency Experiments

**Figure 1:**
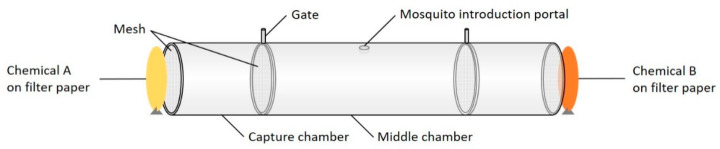
Schematic of the spatial repellency cylinder. Length of the cylindrical chamber, 52 cm; length of each capture chamber, 12 cm; inner diameter, 10 cm; distance between filter paper and capture chamber, 1 cm.

The spatial repellency assay used in the present study was based on the World Health Organization (WHO) guidelines for efficacy testing of spatial repellents [23] and was conducted in a repellency assay cylinder, shown in Figure 1. A two-choice assay was performed to compare the repellency of different types of limonene. In each experiment, two circles of filter papers (90 mm in diameter, Whatman, from Sigma-Aldrich) treated with different types of limonene, providing the same limonene mass (0.82 g), were placed vertically at each end of the spatial repellency assay cylinder, 1 cm away from the end (Figure 1). Concentrations of reagents were chosen based on pilot experiments with a range of R-limonene concentrations. For R-limonene, S-limonene, and ethanol, 1 mL of the pure reagents and for natural orange oil, 3.2 mL of pure orange oil, were added to the filter paper circles (providing the same mass of limonene on filter papers). Then, 20 adult female mosquitoes (aged 5–7 days, starved for 24 h and not blood fed) were added to the middle section of the repellency assay cylinder, via the mosquito introduction portal (Figure 1). Mosquitoes were transferred directly from colony cages to the assay cylinder using a mouth aspirator. After 1 min (as a settlement period), the gates, located in the linking sections of the cylinder, were simultaneously opened. After 10 min, the gates were simultaneously closed. The number of adult mosquitoes in each chamber (including the middle section) was counted. 

In these experiments, the following comparisons were performed: (i) natural orange oil and control, (ii) R-limonene and control, (iii) R-limonene and natural orange oil (which is predominantly R-limonene), and (iv) R-limonene and S-limonene. For each set of comparisons, between 6 and 8 replicates were performed. Ethanol was used as the control in these experiments and the positions of the filter paper circles impregnated with the treatment or ethanol in the repellency assay cylinder were alternated between replicates. Chambers were cleaned with ethanol and left to dry between each replicate. For statistical analysis, we used two-tailed paired *t*-tests in IBM SPSS Statistics version 26 to determine if there were significant differences (*p* < 0.05) between proportions of mosquitoes in each capture chamber. Mosquitoes that were damaged or killed during handling were excluded from the analysis.

## 3. Results and Discussion

### 3.1. Orange Oil, R-Limonene and S-Limonene Compounds

Results from the GC/MS analysis of R- and S-limonene reagents and the cold-press orange oil are provided in Supplementary Table S1. According to the data, limonene is the dominant VOC in all three samples. The chemical compositions of the R- and S-limonene reagents were nearly identical and differed from that of the cold-press orange oil. However, the concentrations of R-limonene in the reagent and R-limonene in the cold-press orange oil were equivalent for the experiment.

### 3.2. Repellency Experiments 

**Figure 2:**
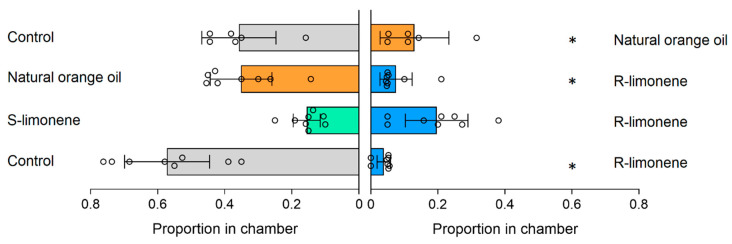
Repellency of different types of limonene in the spatial repellency assays. Bars and error bars represent means and 95% confidence intervals, with circles showing results from individual replicate trials. * denotes a significant repellent effect of the reagent (paired *t*-test *p* < 0.05).

Results of the repellency experiments are provided in Figure 2 and in Supplementary Tables S2–S5. In the experiment comparing R-limonene and natural orange oil, a greater proportion of mosquitoes were captured in chambers with natural orange oil, indicating that R-limonene is more repellent than natural orange oil (*p* = 0.0009, paired two-tailed *t*-test). In addition, in the experiment comparing ethanol with both R-limonene and natural orange oil, a greater proportion of mosquitoes were captured in chambers with ethanol, indicating that both R-limonene and natural orange oil are more repellent than ethanol (*p* = 0.00002, and *p* = 0.03, respectively, paired two-tailed *t*-test). Moreover, in the experiment comparing R-limonene and S-limonene, no significant difference was found between the proportion of mosquitoes captured in chambers, indicating that R-limonene and S-limonene are not significantly different in their repellency (*p* = 0.4, paired two-tailed *t*-test). In addition, the findings show the utility of assays using mosquitoes in identifying differences in biological activity triggered by chemicals isolated from different sources. Previous studies [18,19,20,21] have shown that mosquitoes can be useful and sensitive indicators of toxicity, contamination, and environmental change. Our study suggests the need for further investigation of the effects of synthetic sources of chemicals relative to natural sources, and the potential for the use of mosquitoes as bioindicators.

### 3.3. Limitations

This analysis focused on limonene as the main components of orange oil, R-limonene, and S-limonene. While other components were at minor concentrations (less than 5%), they could pose potential effects on the results. In addition, this study employed mosquitoes as a test species, with repellency as the primary biological effect. Further study is needed to identify specific and direct effects on human or environmental health.

## 4. Conclusions

This study indicates that the same chiral chemical can elicit different biological effects depending on its source. Specifically, a synthetic source of R-limonene was more repellent than a natural source of R-limonene, even when both have the same concentrations. This study provides a foundation for further exploration in possible differences among forms and sources of chiral fragrance chemicals, with implications for air quality and health.

## Data Availability

All data generated or analysed during this study are included in this published article and its supplementary information files.

## Supplementary Materials

The following are available online at https://www.mdpi.com/article/10.3390/ijerph181910505/s1, Table S1. Main VOCs present in the S- and R-limonene reagents and in the cold-press orange oil, Table S2. Repellency rates of adult mosquitoes in the choice test between R-limonene and natural orange oil, Table S3. Repellency rates of adult mosquitoes in the choice test between R-limonene and ethanol, Table S4. Repellency rates of adult mosquitoes in the choice test between natural orange oil and ethanol, Table S5. Repellency rates of adult mosquitoes in the choice test between R-limonene and S-limonene.
