# Supplementary material for: Limonene Emissions: Do Different Types Have Different Biological Effects?
Source: Int J Environ Res Public Health. 2021 Oct 7;18(19):10505. doi: 10.3390/ijerph181910505 (PMC8507918; doi:10.3390/ijerph181910505)
Supplement: Supplementary file 1 [file ijerph-18-10505-s001.zip › ijerph-1375611-supplementary.pdf]

Supplementary Tables

Supplementary Table S1. Main VOCs present in the S- and R-limonene reagents and in the cold-press orange oil.

Compounds	*CAS#*	*R-limonene*	*S-limonene*	*Natural orange oil*
Limonene*	138-86-3	✓	✓	✓
Limonene oxide	1195-92-2	✓	✓	
Acetone*	67-64-1	✓	✓	✓
L-dihydrocarvyl acetate	20777-49-5	✓	✓	
Acetaldehyde*	75-07-0	✓	✓	
beta-trans-Ocimene	3779-61-1	✓		✓
Hydroxyacetone	116-09-6	✓	✓	
beta-Myrcene	123-35-3	✓		✓
D-carvone*	2244-16-8	✓	✓	
4-Isopropenyl-1-methyl-2-cyclohexen-1-ol	7212-40-0	✓	✓	
2-(4-Methylenecyclohexyl)-2-propen-1-ol	29548-13-8	✓	✓	
Perilla alcohol	536-59-4	✓	✓	
Sabinene	3387-41-5	✓		✓
cis-p-Menth-2,8-dienol	22771-44-4	✓	✓	
trans-Carveol	1197-07-5	✓	✓	
Methanol*	67-56-1	✓	✓	
3-Methylhexane*	589-34-4	✓		
L-carveol, mixture of cis and trans	99-48-9	✓	✓	
3-Cyclohexene-1-carboxaldehyde	100-50-5		✓	
1-p-Menthene	5502-88-5		✓	
(Z)-beta-ocimene	3338-55-4			✓
Octanal	124-13-0			✓
beta-Phellandrene	555-10-2			✓
alpha-Phellandrene	99-83-2			✓
Ethanol*	64-17-5			✓
Terpinolene	586-62-9			✓

Linalool	78-70-6	✓
Decanal	112-31-2	✓
alpha-Pinene	80-56-8	✓
(E)-citral*	141-27-5	✓
1-Octanol*	111-87-5	✓
(R)-(+)-citronellal	2385-77-5	✓
beta-Citral*	106-26-3	✓

*Classified as hazardous under Safe Work Australia, Hazardous Chemical Information System (SWA 2021)

Supplementary Table S2. Repellency rates of adult mosquitoes in the choice test between R-limonene and natural orange oil.

		Number of mosquitoes on each side		
Replicates	*Filter paper position (R-limonene - Natural orange oil)*	*R-limonene*	*Natural orange oil*	*Middle*
1	left-right	1	3	17
2	right-left	4	5	10
3	left-right	1	9	11
4	right-left	2	6	12
5	left-right	1	8	10
6	right-left	1	10	11
7	left-right	1	9	10
8	right-left	1	7	12
Average		1.5	7.1	11.6

Supplementary Table S3. Repellency rates of adult mosquitoes in the choice test between R-limonene and ethanol.

		Number of mosquitoes on each side		
Replicates	*Filter paper position (Ethanol - R-limonene)*	*Ethanol*	*R-limonene*	*Middle*
1	left-right	7	1	12
2	right-left	13	1	5
3	left-right	11	0	9
4	right-left	14	1	4
5	left-right	11	1	7
6	left-right	7	1	10
7	right-left	10	0	9
8	left-right	16	1	4
Average		11.1	0.8	8

Supplementary Table S4. Repellency rates of adult mosquitoes in the choice test between natural orange oil and ethanol.

		Number of mosquitoes on each side		
Replicates	*Filter paper position (Natural orange oil - Ethanol)*	*Natural orange oil*	*Ethanol*	*Middle*
1	left-right	3	8	10
2	right-left	2	8	8
3	left-right	6	3	10
4	right-left	2	8	8
5	left-right	1	7	11
6	right-left	1	7	12
Average		2.5	6.8	9.8

Supplementary Table S5. Repellency rates of adult mosquitoes in the choice test between R-limonene and S-limonene.

		Number of mosquitoes on each side		
Replicates	*Filter paper position (R-limonene - S-limonene)*	*R-limonene*	*S-limonene*	*Middle*
1	left-right	6	3	13
2	right-left	5	3	12
3	left-right	8	4	9
4	right-left	1	3	16
5	left-right	1	5	14
6	right-left	4	2	14
7	left-right	4	2	13
8	right-left	3	3	13
Average		4	3.1	13
